# Medical history: as it was; as it will be

**DOI:** 10.5195/jmla.2020.850

**Published:** 2020-01-01

**Authors:** Stephen J. Greenberg

**Affiliations:** Section Head, Rare Books and Early Manuscripts, History of Medicine Division, National Library of Medicine, Bethesda, MD, stephen.greenberg@nih.gov

## Abstract

Born shortly after World War I in 1919 and living through multiple wars, conflicts, and cultural changes in his ninety-six years, Erich Meyerhoff remained a student of history throughout his long life. He regularly attended the annual meetings of the American Association for the History of Medicine and other history groups such as the Medical Library Association’s History of the Health Sciences well into his nineties. This essay traces how the field of history and historical methods changed during Erich’s life and suggests that he saw history and librarianship as a means for achieving social justice and social equity.

Contrary to popular belief, historians do not always like dates. When historians of the British Isles write of the “Long Eighteenth Century,” a shorthand for the period from 1688 (the “Glorious Revolution”) to 1815 (the battle of Waterloo), it is a sign of their desperation. Events in the real world do not happen in tidy little bundles, ready to be categorized, alphabetized, cataloged, and neatly tucked away for future study. History is the vain, Sisyphean attempt to impose order where none exists, except for the schema that practitioners in the field create long, long after the events under scrutiny actually occurred. When one studies and waits long enough, each accepted schema is rejected in its turn, to be replaced with new ideas with their own limited shelf life.

It follows that, if one is a student of history for long enough, the historiography of one’s youth becomes passé, and a new historiography must be learned. The practitioners will change, as will their interests and their methods. Erich Meyerhoff studied history for a very long time, on two continents, and was quite well aware of how the game had changed. It is the aim of this essay to capture at least a little of the field of history during his long lifetime: who wrote it, what they studied, and what historiographic theories and practices were tucked in their tool kits.

Erich received his primary and secondary school education in Germany. Born in 1919 into the political and social turmoil that followed Germany’s defeat in the First World War, he would have started school in the uneasy days of the Weimar Republic. The standard educational pattern would have been a four-year government-sponsored *Grundschule,* possibly followed by another four-year course, and then on to *Mittelschule* and perhaps university. But the increasing anti-Semitism in Germany would have blocked this path, and Erich’s parents sent him to New York in 1935, the year of the Nuremberg Laws that stripped Jews of their rights as German citizens. He received his bachelor of science degree in social science from the City College of New York (CCNY) in 1943.

The history he would have been taught as a child would have been that of Imperial Germany, seen through the lens of nineteenth century historical positivism, the theory developed by Leopold von Ranke (1795–1886). This was no-nonsense stuff, “history as it really was,” based on the exhaustive examination of primary sources: letters, memoirs, archives; no romantic heroes à la Thomas Babington Macaulay in England or the picturesque Jules Michelet writing in France. Von Ranke’s work was almost anti-theory, as the granularity of his research pulverized similarities that might have led to more over-arching theories of historical change. Von Ranke rejected Hegelian models of social change (a thesis that creates its antithesis, leading to a synthesis) and was himself criticized by Karl Marx for underestimating the role of human agency in such change.

A reaction to this sort of history was already afoot in the years following the war. In the work of Max Weber (1864–1920), German historiography found a new model to interpret individual agency and social change. Weber’s most famous book, *The Protestant Ethic and the Spirit of Capitalism*, published in two parts in 1904–1905, created a new approach to examine how disparate, seemingly unconnected forces in society could interact to change economic and political systems. It was the beginning (along with the work of Emile Durkheim in France) of modern sociology. Moreover, Weber’s concerns were not limited to the academic world. He was a vocal critic of Imperial German policies that led to the Great War, and at the time of his early death in 1920, he was working with the committee that was drafting what would become the constitution of the Weimar. However, Weber was no proponent of the Left in Germany; he criticized the Marxists as well as the Kaiser. And when he wrote on the subject of bureaucracy and its relationship with charismatic leaders (in essays that spanned the war), his opinions hewed to no party line or monocausal approach. His social science was value-free and based upon examination and comparison of multiple levels of inquiry.

Another social scientist whose work was changing German historical thought was Georg Simmel (1858–1918). Simmel’s work also contradicted von Ranke, seeing more general and repeatable patterns in social behavior that operated behind historical facts. In such essays as “The Metropolis and Mental Life” (“Die Großstädte und das Geistesleben,” 1903) and “The Stranger” (“Excurs über den Fremden,” 1908), Simmel posited a new approach to history, informed by sociology and psychology, that dealt with situations unknown and, therefore, unstudied by previous scholars: alienation, the loneliness of the individual in a mass society. As a Jew living in Germany, he was born a stranger, and a conventional academic career was closed to him, although he did correspond with his intellectual peers throughout Wilhelmine Germany, including Weber. Simmel died six weeks before the armistice that ended the First World War, but he would not have been surprised by the political and social upheaval the followed the armistice: the collapse of the German monarchy, the vengeful Treaty of Versailles, the rise and fall of the doomed Weimar Republic, and the creation of the Third Reich.

It is little wonder then that Erich joined the exodus of intellectuals (Jews and non-Jews alike) who fled Germany to New York in the 1930s. Alvin Johnson, president of the New School for Social Research, gathered many of these refugees together as the faculty for what he called “the University in Exile,” later renamed the Graduate Faculty of the New School. This was in 1933. New York was a haven for liberal, often “left-leaning” intellectuals in every academic discipline.

Another sympathetic academic address was CCNY, now the flagship school of the City University of New York (CUNY) and part of the State University of New York (SUNY) system, but back then an independent municipal university, with a tradition of free tuition dating back to its founding as the Free Academy of the City of New York in 1847 by educator and businessman Townsend Harris. It was the first free public institution of higher learning in the United States and was one of the first to admit women, albeit only to its graduate programs in 1930 (women could attend Hunter College, founded in 1870). Its appeal to immigrants was undeniable and long-standing, and by the 1930s, it was a bastion of liberal thought; so much so that it was occasionally branded “The Little Red Schoolhouse.” Erich would have felt at home.

I find that I must inject a note of personal history here. My father was born in New York City the year before Erich, to parents who had fled the pogroms of 1905. Although my father attended Townsend Harris High School, the “prep school” for CCNY, his mother (an Old World lady who spoke only Yiddish in her home) had misgivings about him attending such a “left-wing” university. Instead, he attended New York University, which was not free, but was somewhat less “red” and welcoming to immigrants and their children.

The war interrupted Erich’s studies, but he was quick to return to New York when it was over. Initially, he was a social worker, but after receiving his degree from the Columbia University School of Library Service in 1950, he gravitated to medical libraries and cultivated an interest in the history of medicine. The remainder of this essay is devoted to how that history has changed since that date: who writes it, what their topics are, and who the perceived audience might be.

Initially, I attempted some demographic research to answer the first of these questions, but the dearth of evidence was discouraging. The American Association for the History of Medicine (AAHM), founded in 1925, has some historical membership rosters, but the data that can be derived from them are very limited. It is anecdotally accepted that the history of medicine, as it was traditionally written, was seen as the province of medical doctors themselves. Indeed, much of the AAHM membership in the older rosters self-identifies as medical doctors (MDs). Not all did so, and there are a few doctors of philosophy (PhDs), but not many. Gender is also slippery. Woman are sometimes listed as “Miss” and almost always turn out to have been librarians. Some members use only initials for given names, which is a traditional way for women to mask their gender as was (sadly) needed to get things published. This was hardly limited to historical writings in the health sciences. Judging membership by race is totally impossible. The more recent rosters do not list any demographic or educational data.

However, there is some anecdotal evidence available. Attendance at AAHM meetings over the last quarter century gives the unmistakable impression that gender and racial equity, while not yet fully accomplished, is progressing. New areas of equity—concerning sexual orientation, for example, or the representation of non-Western scholars—are at least being addressed. The days of white male MDs writing about the great accomplishments of dead white male MDs for an audience of other white male MDs would seem to be over.

To some degree, perhaps, there was once a point to this sort of annalistic history, a strict retelling of who invented what and who cured whom. It was medicine’s greatest hits, and it was, intellectually at least, claimed by the doctors. After all, one needed a medical education to fully understand “history as it really was”—Von Ranke all over again. Was it not true that one would be a better physician if one knew the man behind the eponym; for example, Percivall Pott (1714–1788), of Pott’s fracture, Pott’s disease, Pott’s puffy tumor, and so on?

At one level, this is undoubtedly correct, but only if the history of medicine is merely a litany of technological and pharmaceutical triumphs. But it is not only that, nor is it the exclusive property of the doctors themselves. Just as medicine has social responsibilities to the populations its serves, so does its history. If one is not satisfied with any current health care system (and, viewed from any political stance, there are few that ARE satisfied), then one must look to the history of medicine to determine where we are, how we got there, and what might be the way forward.

Perhaps the first to write about this in any detail was the Swiss physician and historian Henry Sigerist (1891–1957). Trained as a physician in Zurich, he emigrated to the United States and directed the Institute of the History of Medicine at School of Medicine from 1932 to 1947. Sigerist, knowingly or not, subscribed to a rather Whiggish school of historiography, where all events move (more or less directly) toward a predetermined goal. In the case of the history of medicine, the goal was universal health care in the form of socialized medicine. These thoughts, espoused in the 1930s and 1940s, had a very sympathetic audience in parts of Canada (Saskatchewan enacted a limited form of state-sponsored health care in 1944) but also earned him the enmity of the American Medical Association. But Sigerist had succeeded in opening a door through which many followed, and the history of medicine increasingly came to be seen as a weapon in the struggle for social justice. The central battlefield has come to be the enormous disparity in available and appropriate health care based on race, gender, and (since the emergence of HIV/AIDS) sexual orientation.

History as a weapon would seem to go against the grain of the historians and sociologists seeking a value-free social science, but in fact, it has always been so. After all, Weber chose to involve himself in the creation of the Weimar Republic, and even von Ranke worked for the Prussian state, editing a state-supported academic journal that attacked liberals and their causes. It is perhaps worth noting here that laboratory scientists got involved as well: the famous German physician, researcher, and polymath Rudolph Virchow (1821–1902) was a long-serving liberal member of the Prussian Diet (later the Reichstag), where he frequently locked horns with the formidable chancellor, Otto von Bismarck. Sigerist’s primary contribution was to move the playing field to the arena of public opinion, in the United States during a period (Roosevelt’s “New Deal”) that saw unprecedented steps toward government involvement in and commitment to social justice. How and why these measures fell short, and what could be accomplished moving forward, are the questions still on the table, and their answers require the input of historians.

Disparity in the delivery of quality health care is probably the single biggest issue that faces the modern health care system at virtually every level. It is certainly the focus of considerable research in all the social sciences, not just among historians. Evidence from specific cases, varying in time and space, continues to mount, but if *any* major medical, social, and/or economic conundrum can be said to have a unitary cause, then surely a straight line can be drawn from the persistent disparities in the delivery of affordable, high-quality health care to systemic racism and sexism. As more research in the history of medicine is made public—done by health care practitioners, social scientists, and other researchers—this line becomes ever more distinct. Using historical knowledge and understanding as a weapon to end that connection is the task for historians moving forward.

Those of us who knew Erich and who spoke with him about his concerns for achieving social justice can have no doubt where he stood on such topics. He was neither an ideologue nor a fanatic, but he knew that history and for that matter librarianship were not ends in themselves but were means to far greater ends: the achievement of social justice and social equity for all, regardless of religion, gender, race, or sexual orientation. A refugee from Nazi oppression and only sixteen years old when he arrived in New York City, he never forgot the history of his home country or his heritage as a Jew. History means identity, remembered and shared, sometimes even treasured, but it is also a springboard to a better, more equitable future for everyone.

Stephen J. Greenberg, MSLS, PhD, AHIP,* stephen.greenberg@nih.gov, Section Head, Rare Books and Early Manuscripts, History of Medicine Division, National Library of Medicine, Bethesda, MD

